# Trained immunity: A Yin‐Yang balance

**DOI:** 10.1002/mco2.121

**Published:** 2022-03-06

**Authors:** Zhidong Hu, Shui‐Hua Lu, Douglas B. Lowrie, Xiao‐Yong Fan

**Affiliations:** ^1^ Shanghai Public Health Clinical Center Key Laboratory of Medical Molecular Virology of MOE/MOH Fudan University Shanghai China; ^2^ National Medical Center for Infectious Diseases of China Shenzhen Third People Hospital, South Science & Technology University Shenzhen China

**Keywords:** immunotherapy, innate immune memory, trained immunity, Yin‐Yang balance

## Abstract

Traditionally, immune memory is regarded as an exclusive hallmark of adaptive immunity. However, a growing body of evidence suggesting that innate immune cells show adaptive characteristics has challenged this dogma. In the past decade, trained immunity, a de facto innate immune memory, has been defined as a long‐term functional reprogramming of cells of the innate immune system: the reprogramming is evoked by endogenous or exogenous insults, the cells return to a nonactivated state and subsequently show altered inflammatory responses against a second challenge. Trained immunity became regarded as a mechanism selected in evolution to protect against infection; however, a maladaptive effect might result in hyperinflammation. This dual effect is consistent with the Yin‐Yang theory in traditional Chinese philosophy, in which Yang represents active, positive, and aggressive factors, whereas Yin represents passive, negative, and inhibitory factors. In this review, we give a brief overview of history and latest progress about trained immunity, including experimental models, inductors, molecular mechanisms, clinical application and so on. Moreover, this is the first time to put forward the theory of Yin‐Yang balance to understand trained immunity. We envision that more efforts will be focused on developing novel immunotherapies targeting trained immunity in the coming years.

## Graphical abstract

Trained immunity, a de facto innate immune memory, is defined as a long‐term functional reprogramming of cells of the innate immune system. The dual effect of trained immunity is consistent with the Yin‐Yang theory in traditional Chinese philosophy. The Yin‐Yang aspects of trained immunity provide an opportunity to treat inflammatory, infectious, and autoimmune diseases by optimizing innate immune memory.

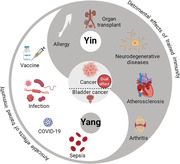

## 1 INTRODUCTION

Trained immunity, a de facto innate immune memory, has been defined as an adaptation of the innate host defense system. The vertebrate immune system has traditionally been divided into two arms, the nonspecific but broad‐coverage innate immune system, and the highly specific memory‐driven adaptive immune system.^1^ Thus, it was assumed that immune memory was an exclusive hallmark of the adaptive immune system for a long time. However, a growing body of evidence suggests that innate immune cells have adaptive characteristics, thus challenging this dogma. In the past decade, trained immunity, initially defined as a heterologous immunological memory in innate immune cells, emerged as a focal point in immunology research and added a layer of complexity to the previous understanding of immune systems. In this review, we first review the discovery process and an updated definition of trained immunity and then introduce different cell subsets that can develop trained immunity in current research models. Then, we provide an overview of trained immunity inducers and their mechanisms. We propose that the effects of both the beneficial and detrimental functions of trained immunity can be seen as compatible with the Yin‐Yang theory in traditional Chinese philosophy, in which Yang represents active, positive, and aggressive factors, whereas Yin represents passive, negative, and inhibitory factors. The Yin and Yang aspects of trained immunity in human health are surveyed. Finally, we summarize the recent advances of trained immunity‐based immunotherapies and provide possible directions for future research in this field.

## 2 AN OVERVIEW OF TRAINED IMMUNITY

### 2.1 The definition of trained immunity

Plants and invertebrate animals lack the adaptive antigen‐specific immune system. However, when plants are inoculated with a specific pathogen, they can gain immunity to this and other pathogens including those never exposed to; this effect was called systemic acquired resistance.^1^, ^2^ Analogously, in invertebrates, macrophages, an innate immune cell subset, can become primed and protect the animal for life against a secondary, otherwise lethal reinfection with the same pathogen.^3^ In humans, several epidemiological studies established that vaccination with Bacille Calmette‐Guérin (BCG), the smallpox vaccine, and others protect infants against heterologous microorganisms.^4^, ^5^, ^6^, ^7^ Thus, plants, invertebrates, and vertebrate animals could develop immune memory in innate immune systems, resulting in nonspecific effector functions of the innate immune cells against heterologous microorganisms.

Netea M. and his group in The Netherlands have been particularly instrumental in establishing the concept of trained immunity and they gave it the name. In research aiming to assess the impact of BCG vaccination on specific antimycobacterial immune responses, they found that elevated immune responses were shown not only against *Mycobacterium tuberculosis* stimulation but also against a nonrelated stimulus, *Candida albicans*. After further experimental validation and a survey of an extensive background of published observations consistent with the existence of the phenomenon, their landmark perspective was published in 2011^8^ and updated in 2017.^9^ The definition has been further refined as being the long‐term functional reprogramming of the innate immune cells that are evoked by endogenous or exogenous insults, with the cells returning to a nonactivated state, yet showing altered inflammatory responses (IL‐1β, IL‐6, and TNF‐α) and protection against a second homologous/heterologous challenge.^10^

### 2.2 The difference and association between trained immunity and adaptive immunity

The de facto innate immune memory that is trained immunity is different from the immune memory in adaptive immunity. First, trained immunity is dominantly mediated by myeloid cells, whereas adaptive immune memory is a unique characteristic of lymphocytes, mainly T cells and B cells. Second, adaptive immunity is initiated by a process of combining of peptide‐MHC (pMHC) complexes with T/B cell receptors (TCR/BCR), which takes days or even weeks to become established. After the clearance of invading pathogens, some effector cells are transformed into long‐lived memory cells. In contrast, trained immunity is primed by the binding of pattern recognition receptors and generic molecules which are produced by microbes or other inducers, and it only takes a few hours or even minutes to get activated. After removal of the stimulus, an “epigenetic scar” on the stimulated genes can persist in these relatively short‐lived trained innate immune cells. Third, the immune memory of adaptive immunity is antigen‐specific, robust, and long‐lasting, whereas the innate immune memory is nonspecific, modest, and exists for a relatively short time.

To be noted, the dichotomy between the adaptive and innate immune responses is actually an academic simplification. In the past decades, these two halves of the immune system have been shown to be interlaced with each other. The concept of trained immunity in innate lymphoid cells (ILCs), developed with the finding that natural killer (NK) cells (a classic innate cell subset) also possess adaptive immune characteristics^11^ blurring the distinction between the two immune systems. Trained immunity and adaptive immunity can share mechanisms. On the one hand, the enhanced production of inflammatory cytokines induced from trained innate immune cells might amplify the downstream effects of lymphocyte activation by providing the “third signal” besides the signal from pMHC‐TCR/BCR interaction and the costimulatory signal from accessory molecules. Thus, during a secondary challenge, the microenvironment constructed by trained immunity might modify the specific outcome of cellular and humoral immune responses by altering the differentiation and polarization of lymphocytes. An example of this may be when a fungal infection trains dendritic cells (DCs), the specialized antigen‐presenting cells, which then participate in the modulation of adaptive immunity.^12^ On the other hand, adaptive immune cells, such as effector CD8 lymphocytes, are indispensable for the homing of trained innate cells to the respiratory mucosa in the early stage of viral infection^13^ and this provides the first example of reverse signaling from an adaptive immune response for the induction of trained immunity. Thus, it will be crucial to further investigate the interaction between trained and adaptive immunity, so that novel immunotherapies and vaccines can be devised to simultaneously optimize the two halves of the immune system; this prospect will be described in Section 6 of this review.

### 2.3 The cell subsets that can develop trained immunity

**FIGURE 1:**
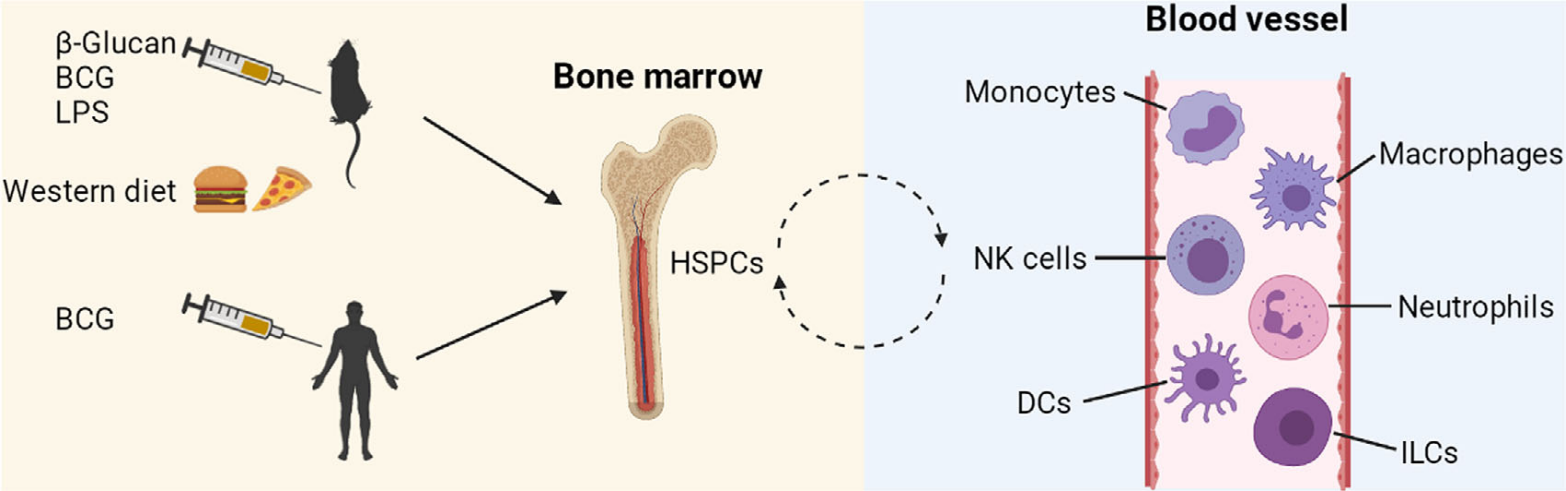
Long‐term myelopoiesis reprogrammed in HSPCs that subsequently differentiate into several cell subsets. The elevated trained immunity that is observed at least 1 year after BCG vaccination indicates that long‐term changes in myelopoiesis exist in addition to the short‐term changes in circulating mature myeloid cells. β‐glucan, BCG, LPS, and western diet induce reprogramming of myeloid progenitor cells in the bone marrow. HSPCs continually undergo asymmetric division in the bone marrow and provide a repertoire of myeloid and lymphoid cell subsets. These subsets circulate through the body and can reside in different tissues. The cell subsets include monocytes, macrophages, NK cells, neutrophils, DCs, and ILCs. These processes provide the basis for short‐lived innate immune cells acquiring memory with persistent phenotypes in vivo. BCG, Bacille Calmette‐Guérin; DCs, dendritic cells; HSPCs, hematopoietic stem and progenitor cells; ILCs, innate lymphoid cells; LPS, lipopolysaccharide; NK, natural killer. This figure was created with BioRender.com

The initial studies of trained immunity were mainly focused on monocytes, macrophages, and hematopoietic stem and progenitor cells (HSPCs), which will be described in the next section. However, recent studies have shown that trained immunity can also be induced in other cell subsets such as NK cells, DCs, neutrophils, and ILCs (Figure 1).

NK cells are an important part of the innate immune system, and were among the first shown to develop trained immunity; NK cells isolated from BCG‐immunized volunteers showed a trained immunity profile after ex vivo stimulation with unrelated pathogens.^14^, ^15^ More recently, it was shown that repeated pregnancies generated a unique transcriptome and epigenetic signature in decidual NK cells; the trained cells showed increased secretion of VEGF‐α and IFN‐γ, and constituted a trained memory of pregnancy to better support subsequent pregnancies.^16^ DCs are important components of the innate immune system that bridge innate and adaptive immune responses. They are traditionally considered to lack antigen specificity and to be devoid of immunological memory. However, as mentioned above, DCs isolated from mice that had been immunized by fungal infection exhibited long‐term memory‐like cytokine recall responses upon subsequent challenge with the fungal pathogen. Abrogation with inhibitors of specific histone modifications suggested a trained immunity phenotype.^12^ Neutrophils are another crucial component of the innate immune response, providing the first line of defense against invading pathogens. They have been demonstrated to participate in trained immunity, too. A β‐glucan‐induced antitumor effect was associated with epigenetic and transcriptomic reprogramming of granulopoiesis and neutrophils were “rewired” to acquire an antitumor profile; neutrophils that were adoptively transferred from β‐glucan‐treated mice suppressed tumor growth.^17^ In humans, BCG vaccination induced long‐term immunophenotypic changes in neutrophils and increased neutrophil‐dependent antimicrobial activity against unrelated pathogens.^18^ There is also evidence that ILCs, which function to bridge between innate and adaptive immunity, might also acquire trained immunity.^10^

### 2.4 Experimental models of trained immunity

**FIGURE 2:**
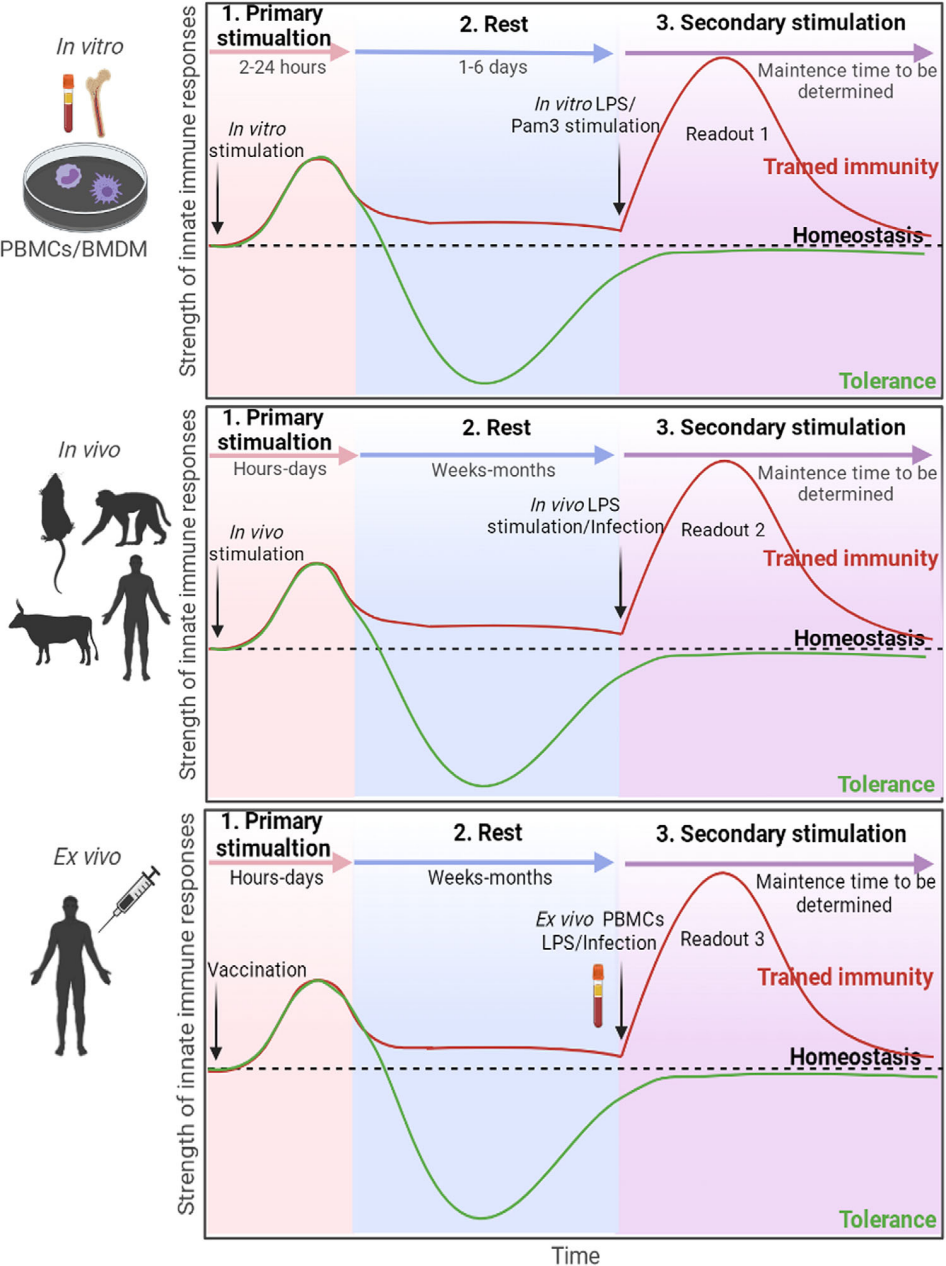
Experimental models to evaluate trained immunity in vitro, in vivo, and ex vivo. The in vitro model mainly uses human PBMCs‐derived monocytes and mouse bone marrow‐derived macrophages. The cells are trained with inducers such as β‐glucan and BCG, and then rested without stimulation for several days to return to steady states. The cells are restimulated with LPS or Pam3Cys for 24 h. The production of IL‐1β, IL‐6, and TNF‐α in the supernatant is evaluated, and the epigenetics and metabolism of trained cells are determined. In the in vivo model, the animals are vaccinated or stimulated with inducers of trained immunity. After a resting period, the animals are restimulated with LPS or challenged with heterologous pathogens. Protection against infection is evaluated in addition to the cytokines, metabolic and epigenetic changes. Protection against heterologous infection in humans can also be assessed in follow‐up trials. In the ex vivo model, PBMCs are taken from the vaccinated volunteers, to evaluate trained immunity similarly to the in vitro models. The curves in black represent the homeostasis of the innate immune system; the curves in red represent the trained immunity, in which the “trained” innate cells‐induced responses were enhanced after 2nd stimulation; the curves in green represent immune tolerance, in which the 2nd responses were suppressed. To be noted, the ceiling capacity of trained immunity induction in different experimental models remains to be elucidated and direct comparison of the values of trained immunity in different models is not appropriate. Readout 1: Epigenetics, metabolism, and IL‐1β/IL‐6/TNF‐α’s detection in the supernatant of the cell culture medium. Readout 2: Epigenetics, metabolism, IL‐1β/IL‐6/TNF‐α’s detection in the serum, and protection against heterologous infection. Readout 3: Epigenetics, metabolism, and IL‐1β/IL‐6/TNF‐α’s detection in the serum. BMDMs, bone marrow‐derived macrophages; LPS, lipopolysaccharide; PBMCs, peripheral blood mononuclear cells. This figure was created with BioRender.com

The first published experimental model of trained immunity used human peripheral blood mononuclear cells (PBMCs) and/or sorted monocytes. The cells were stimulated (trained) with β‐glucan, heat‐killed *C. albicans*, mannans, or *Escherichia coli* for 24 h. The cells were then rested in the culture medium without any stimulation for 6 days to return to a steady‐state. Subsequently, cells were restimulated for 24 h with various stimuli such as lipopolysaccharide (LPS) and TLR2 agonist Pam3Cys. The supernatants were collected and tested for the production of inflammatory cytokines. The cells showed enhanced responses to heterologous secondary stimuli if the primary training was effective.^19^ This model was further optimized by using different durations of training and resting phases^20^ and has been adapted as an in vitro model with mouse‐derived cells.^21^, ^22^ In the in vitro mouse cell model, bone marrow‐derived macrophages (BMDMs) were exposed to β‐glucan for 24 h and rested for 3 days without any stimulation. The cells were then primed with IFN‐γ (which was required to detect LPS‐induced cytokines) and stimulated with LPS 24 h later. The secretion of IL‐1β was determined 6 h later upon addition of adenosine triphosphate (ATP, which was needed for inflammasome activation and pro‐IL‐1β processing), and the production of IL‐6/TNF‐α was assayed after 24 h of LPS stimulation.^21^ Such in vitro models are currently regarded as standard procedures of trained immunity evaluation (Figure 2).

In in vivo mouse models, two aspects were considered: the peripheral cells and bone marrow progenitors. The model for peripheral cells utilized normal C57BL/6 mice or SCID mice which lack the adaptive immune system. The mice were vaccinated with BCG or another TB vaccine, MTBVAC. Four weeks later, the mice were stimulated with LPS as a heterologous stimulation, and the production of IL‐1β, IL‐6, and TNF‐α in the serum was determined after 4 h. Alternatively, the vaccinated mice were infected with heterologous *Streptococcus pneumoniae* 9 weeks later and nonspecific protection against infection was evaluated.^23^ The progenitor cell model used parabiotic and chimeric mice for adoptive cell transfer to determine if intravenous administration of BCG “educated” HSPCs for heterologous protection.^24^, ^25^ Assays for in vivo‐trained immunity evaluation were also established in other animal models such as rhesus macaques^26^ and calves.^27^ Furthermore, a recent randomized clinical trial of BCG vaccination in the elderly showed immune protection against heterologous infections and improved survival independently of tuberculosis prevention,^28^ and an investigator‐blind randomized controlled trial showed that BCG vaccination at birth protected against significantly reduces all‐cause infectious disease morbidity during the neonatal period.^29^ This could be regarded as an in vivo evaluation model of vaccine‐induced trained immunity (Figure 2).

An ex vivo human model of trained immunity has also been established. In this model, PBMCs that are collected from “naive” volunteers before and after BCG vaccination are stimulated with homologous and heterologous stimuli, and the secretion of inflammatory cytokines is assayed^30^ (Figure 2). However, there are several limitations of this ex vivo model compared with murine models due to ethical and safety concerns. Furthermore, unlike laboratory mice, human beings undergo continually varying challenges to their immune systems through their changing environments and have diverse genetic backgrounds. These are important confounding factors when evaluating novel vaccines/agonists through this model. Nevertheless, this human model provides an important supplement to trained immunity research.

## 3 INDUCERS OF TRAINED IMMUNITY

### 3.1 β‐glucan

As mentioned above, the fungal pathogen *C. albicans* and its cell wall component β‐glucan were among the first agonists found to induce in vitro‐trained immunity.^19^ The in vivo training effect of β‐glucan was first described after intraperitoneal administration to mice. This treatment enhanced myelopoiesis and induced a favorable response to a secondary challenge that protected the trained mice from chemotherapy‐induced myelosuppression.^31^ Zymosan is a cell wall preparation rich in β‐glucan and mice trained with zymosan acquired broad‐spectrum protection against lethal infections of several kinds of bacteria, which was associated with modulation of IL‐1 signaling and bone marrow hematopoietic progenitors.^32^ β‐glucan is now one of the most frequently used inducers in experimental studies in the field of trained immunity.

### 3.2 BCG vaccine

As illustrated above, the first report of BCG‐induced trained immunity had shown that BCG vaccination in healthy volunteers enhanced the release of monocyte‐derived cytokines in response to unrelated bacterial and fungal pathogens, and induced lymphocyte–independent protection of immunodeficiency SCID mice against disseminated candidiasis.^30^ These effects remained for at least 1 year after vaccination,^33^ and were accompanied by enhancement of glycolysis and epigenetic regulation of monocytes.^34^ Furthermore, in a randomized placebo‐controlled study of response to an experimental viral challenge, an enhancement of heterologous protection induced by BCG was correlated with up‐regulation of IL‐1β, a marker of trained immunity.^35^ Interestingly, BCG that had been killed by γ‐irradiation retained the ability to induce trained immunity, suggesting this effect was mediated by structures rather than products of the live bacterium.^36^ Although most bacteria produce N‐acetyl muramyl dipeptide (MDP), mycobacteria produce N‐glycolyl MDP, and this is more potent in inducing innate immunity^37^, ^38^; the training effects induced by BCG could be reproduced, at least partially, with MDP,^30^, ^39^ consistent with the involvement of MDP in BCG‐induced trained immunity. However, in comparison to BCG, *M. tuberculosis* infection showed reduced induction of protective trained immunity and this was associated with impaired IFN‐1 signaling.^25^ This suggests an interplay of agonists and antagonists in mycobacterium‐induced trained immunity besides any effect of MDP.

### 3.3 Other inducers

Other infective agents besides BCG and *M. tuberculosis* are natural inducers of trained immunity. Respiratory infection with an adenovirus was found to induce alveolar macrophages that had a long‐lasting memory, sustained by an enhanced trained immunity phenotype in the local mucosal sites.^13^ This could result in robust host defense against heterologous bacterial infection. The trained memory macrophages required CD8 T cell help in the priming phase but were then self‐sustaining in the alveoli and independent of circulating monocytes and T cells. They displayed properties of trained immunity such as a defense‐ready signature and high rates of glycolysis.^13^ Human immunodeficiency virus‐infected people showed increased levels of IL‐1β production in responses to LPS and bacterial infection.^40^ Hepatitis B virus exposure in utero triggered a trained immunity state, characterized by enhanced innate immune activation, which increased the ability of cord blood immune cells to respond to bacterial infection.^41^ Innate immunity could also be trained by *Borrelia burgdorferi* ^42^ and *Plasmodium falciparum*.^43^ Vaccines such as inactivated diphtheria/tetanus/whole‐cell pertussis vaccine^44^ and tuberculosis vaccine MTBVAC^23^ showed classic trained immunity characteristics in mouse immunization models; they conferred heterologous protection against infection. Thus, a broad range of infection and vaccine types can induce trained immunity. In addition, oxidized low‐density lipoprotein (oxLDL) also induced trained immunity through the glycolytic pathway.^45^, ^46^ Endotoxin, the LPS of Gram‐negative bacteria, can be considered as an agonist of trained immunity. At extremely low doses it can prime the immune system for a more vigorous response to secondary challenges.^47^ However, people and animals undergo continuous environmental exposure to large doses and that necessitates the development of tolerance. Indeed, primary contact of monocytes with high doses of LPS drives them into a tolerant state during which they are refractory to subsequent stimulations.^47^

## 4 MECHANISMS OF TRAINED IMMUNITY

Induction of trained immunity appears to be mediated by long‐term adaptations in the chromatin of innate immune cells that render the DNA more accessible to the transcriptional machinery.^48^ The reciprocal effects of the resulting epigenetic and metabolic reprogramming in myeloid cells and HSPCS that modulate trained immunity are not yet fully understood.

### 4.1 Immunometabolic circuits

**FIGURE 3:**
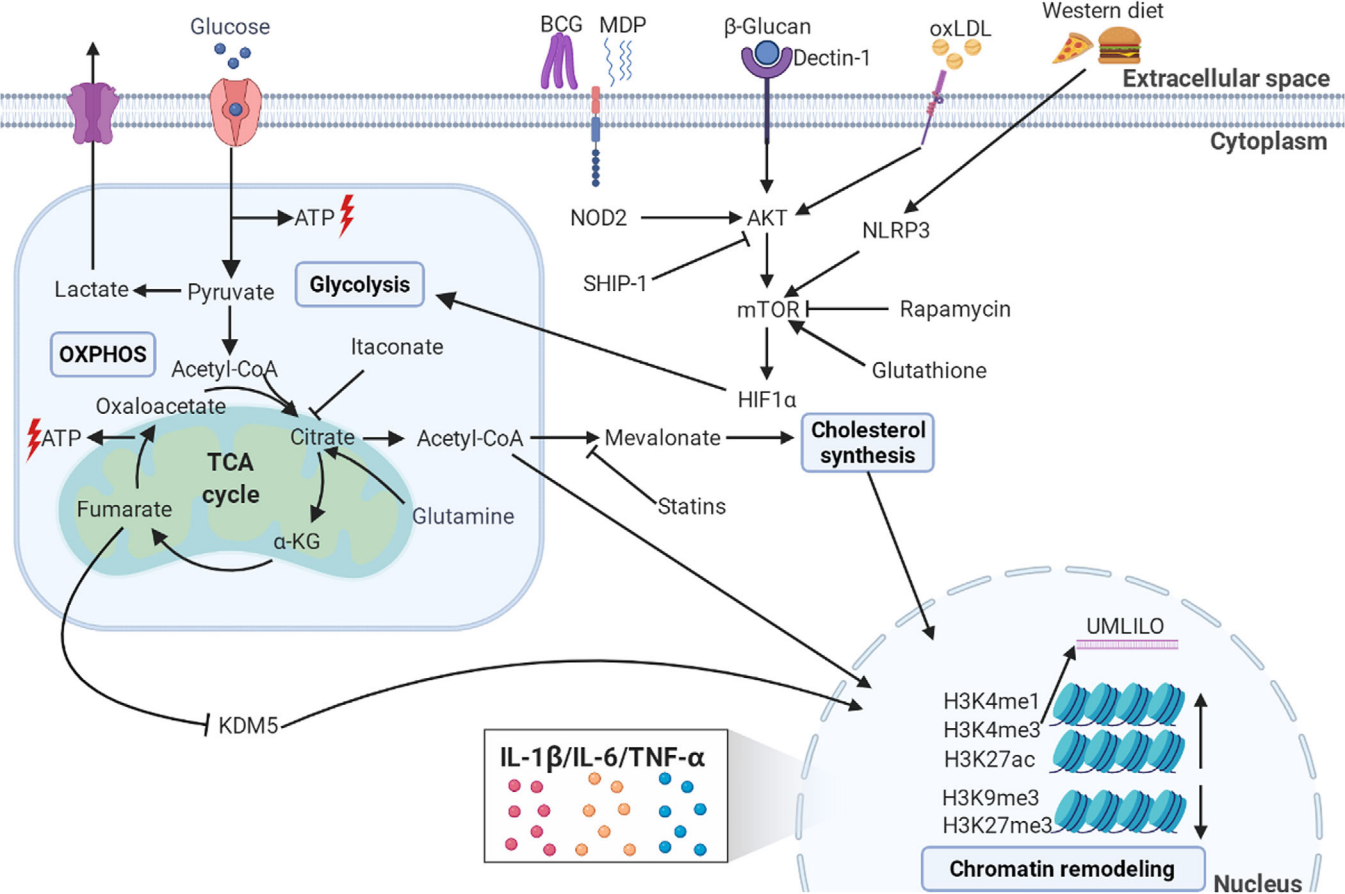
Mechanisms of trained immunity. The trained immunity stimuli such as β‐glucan, BCG, MDP, and oXLDL enter the cell cytoplasm through diverse receptors, then activate the Akt‐mTOR‐HIF1α axis to modulate metabolic reprogramming including glycolysis‐OXPHOS and the TCA cycle. These processes can be impaired through inhibitors like rapamycin and SHIP‐1. Certain metabolites such as acetyl‐CoA and fumarate derived from these enzymic processes can, in turn, mediate epigenetic remodeling of histones; for example, by inhibiting lysine demethylase KDM5 and increasing histone methylation and acetylation at genes involved in the innate immune responses. The remodeling leaves epigenetic markers such as H3K4me1, H3K4me3, and H3K27ac. Acetyl‐CoA can also modulate the cholesterol synthesis pathways through mevalonate, and inhibitory statins can suppress this process. α‐KG, α‐ketoglutarate; ATP, adenosine triphosphate; BCG, Bacille Calmette‐Guérin; MDP, muramyl dipeptide; mTOR, mammalian target for rapamycin; oxLDL, oxidized low‐density lipoprotein; OXPHOS, oxidative phosphorylation; TCA, tricarboxylic acid cycle. This figure was created with BioRender.com

Metabolites in several metabolic circuits have been reported to be involved in training innate immune cells. In 2014, transcriptomes and epigenomes of β‐glucan/dectin‐1‐trained macrophages were shown to have an exclusive metabolic and epigenetic signature that suggested a connection between metabolic pathways and epigenetic reprogramming.^49^ In the same year, it was reported that changes in cellular metabolism, with a shift from oxidative phosphorylation (OXPHOS) to aerobic glycolysis (also known as the Warburg effect) through an Akt‐mTOR‐HIF1α pathway, was crucial for the induction of β‐glucan‐induced trained immunity.^50^ BCG and its bioactive peptide MDP triggered in this process via the NOD2 receptor, and a western diet mediated the trained immunity through NLRP3.^51^ Metabolic profiles indicating glycolysis upregulation were also observed in oxLDL‐trained innate immune cells.^45^, ^46^ More recently, it was found that impaired glutathione generation compromised mTOR activation, and suppressed β‐glucan‐induced proinflammatory cytokine production and protection against heterologous second challenge.^52^ During this process of glycolysis activation, rapamycin might inhibit the mTOR pathway in a dose‐dependent manner to suppress the training effect.^50^ In addition, SHIP‐1‐deficient macrophages exhibited increased phosphorylation of Akt, and this correlated with augmented glycolytic metabolism; inhibition of SHIP‐1 could enhance trained immunity.^21^ These data suggested that the elevation of aerobic glycolysis through the Akt‐mTOR‐HIF1α axis is the metabolic basis of trained immunity, providing the energy and the necessary substrates for the activation of innate immune cells. Meanwhile, several antagonists have been identified that suppress trained immunity induction (Figure 3).

The downstream signaling pathways of glycolysis involved in trained immunity are beginning to be understood. Upregulation of glycolysis leads to the increased uptake of glucose, which is converted into pyruvate and transformed into lactate that is subsequently released from the cell, instead of entering the mitochondria to undergo oxidation.^53^, ^54^ Subsequently, the tricarboxylic acid (TCA) cycle uses the energy from acetyl‐CoA and transfers it to OXPHOS, which results in ATP production. Several TCA intermediates were reported to be involved in the tuning of innate immune memory. Citrate could either be produced from glycolysis from pyruvate or be derived from other metabolites such as glutamine, which could be converted into α‐ketoglutarate (α‐KG) to enter into the TCA cycle. Glutamine replenishment resulted in the accumulation of fumarate via citrate and α‐KG, and this integrated the immune and metabolic circuits to induce monocyte epigenetic reprogramming by inhibiting KDM5 histone demethylases.^53^ The production of α‐KG via glutaminolysis could orchestrate macrophage activation via metabolic changes and epigenetic reprogramming mediated by the demethylase JMJD3 and fatty acid oxidation.^55^ In contrast, itaconate could induce immune tolerance through alkylation of cysteine residues on the protein KEAP1, thereby allowing the anti‐inflammatory transcription factor NRF2 to increase the expression of anti‐inflammatory genes^56^; β‐glucan could counteract tolerance induction by inhibiting the expression of immune‐responsive gene 1, an enzyme that controls itaconate synthesis.^57^ The metabolite mevalonate, an essential mediator in the cholesterol synthesis pathway, induced trained immunity via activation of the insulin‐like growth factor 1 receptor and mTOR signaling, blockage of the process by 3‐hydroxy‐3‐methylglutaryl coenzyme A reductase inhibitory statins prevented trained immunity induction; Monocytes of patients with hyperimmunoglobulin D syndrome who were deficient in mevalonate kinase had a constitutive trained phenotype, which could explain the attacks of sterile inflammation that these patients experience.^58^ Enhanced cholesterol synthesis was also observed in β‐glucan trained HSPCs, and this was associated with the accumulation of cholesterol esters and lipids with more‐saturated acyl chains^31^ (Figure 3).

To sum up, the rewiring of cellular metabolic pathways, including glycolysis, OXPHOS, and cholesterol synthesis, were found to be the critical mediators of trained immunity modulation. Among these, the Akt/PI3K/mTOR pathway and the TCA cycle appear to be the common denominators; their interaction leads to the regulation in histone acetylation and methylation in the promoters and enhancers of genes encoding inflammatory cytokines, which will be described below.

### 4.2 Epigenetic reprogramming

The metabolic reprogramming seen in trained innate immune cells is accompanied by changes in the epigenetic landscape. After first sensing a pathogenic microorganism through pattern recognition receptors, monocytes are rapidly activated and initiate downstream transcriptional cascades, which are tightly regulated at the chromatin level. Dynamic transcription patterns are generated containing a broad range of effector molecules.^59^ Epigenetic regulators are prominently involved in these cascades. After removal of the stimulus, an “epigenetic scar” on the stimulated genes can persist, changing the long‐term responsiveness to secondary stimulation and manifesting as a functional trained immunity program.^60^ Epigenetic regulation is mainly determined by posttranslational histone modifications, DNA methylation, chromatin remodeling, and the production of long noncoding RNAs (lncRNAs).

The epigenetic markers that participate in trained immunity regulation were identified as histone 3 lysine 4 methylation (H3K4me1),^49^ which marks distal enhancers; histone 3 lysine 4 trimethylation (H3K4me3),^19^, ^30^ which marks active promoters of stimulated genes; histone 3 lysine 27 acetylation (H3K27ac),^49^ which marks active enhancers and promoter regions; and histones H3K9me3^34^ and H3K27me3,^61^ which are reduced during training. Regarding the contributions of DNA methylation and lncRNAs to the epigenetic reprogramming, analyses of global DNA methylation patterns in responders and nonresponders to BCG vaccination showed a stable and robust differential DNA methylation pattern among the promoters of genes belonging to immune pathways in responders. Responders were defined on the basis of enhanced macrophage capacity to restrict the growth of *M. tuberculosis* accompanied by a higher level of IL‐1β production.^62^ A follow‐up study identified 43 differentially methylated genes present before vaccination that could be used as possible predictors of responsiveness to stimuli that induce trained immunity.^63^ LncRNAs were identified as functional modulators of gene expression that are directed to specific loci by the spatial organization of the chromatin.^64^ The β‐glucan‐mediated training of human monocytes increased the levels of H3K4me3 bound to UMLILO, a lncRNA‐regulated immune gene promoter; the resultant inhibition of UMLILO prior to training significantly decreased chemokine transcription.^65^ In addition, the impaired glutathione synthesis in β‐glucan‐trained macrophages decreased the H3K27me3 demethylation in the promoters of immunometabolic genes by raising the level of the methyltransferase enhancer of zeste homolog 2; inhibition of zeste homolog 2 amplified trained immunity‐mediated heterologous immune protection.^52^ The metabolic rewiring and epigenomic reprogramming of innate immune cells and their progenitors that underlies trained immunity are summarized in Figure 3.

### 4.3 Reprogramming of HSPCs

Trained immunity was first described based on the nature of mature myeloid cells such as monocytes and macrophages. However, elevated trained immunity was found present at least 1 year after BCG vaccination,^33^ indicating that long‐term change in myelopoiesis was occurring through reprogramming of HSPCs. In 2018, two research groups independently showed that trained immunity occurred in bone marrow progenitor cells.^24^, ^31^ By using parabiotic and chimeric mice, as well as adoptive transfer animal models, Kaufmann et al. showed that the intravenous administration of BCG “educated” HSPCs so that they subsequently generated epigenetically modified macrophages that provided heterologous protection.^24^ Mitroulis et al. showed in mice that β‐glucan induced in vivo expansion of HSPCs that utilized glucose metabolism and cholesterol biosynthesis pathways. The resultant cells provided a beneficial response to secondary LPS challenge and protection from chemotherapy‐induced myelosuppression.^31^ Further investigation showed, in a murine HSPCs transplantation model, that the dectin‐1‐MyD88‐TLR2 axis was crucial for β‐glucan‐induced trained immunity.^66^ The induction of trained immunity in mice that was caused by switching to a western diet was also associated with epigenomic reprogramming of myeloid progenitor cells.^51^ Transient stimulation of mice with LPS induced persistent C/EBPβ‐dependent alterations in the transcription accessibility among some specific myeloid lineage enhancers in hematopoietic stem cells and this resulted in increased responsiveness of the associated immune genes upon secondary infection.^67^ In healthy human volunteers, intradermal BCG vaccination induced trained immunity, which was mediated by a persistent transcriptomic myeloid bias in HSPCs in the bone marrow. Persistent epigenetic changes in peripheral CD14^+^ monocytes resulted.^68^ Recently, it was found that extracellular “labile” heme, released from damaged red blood or parenchymal cells, could train innate immunity in HSPCs and promote resistance against sepsis; the training depended on the splenic tyrosine kinase signal transduction pathway acting upstream of c‐Jun N‐terminal kinase.^69^ These studies together provide some indication of how short‐lived innate immune cells can acquire memory with a persistent phenotype in vivo (Figure 1).

### 4.4 Other factors associated with trained immunity

As mentioned above, diet and lifestyle can also modulate trained immunity. Feeding a western diet to mice resulted in a systemic NLRP3‐dependent inflammation profile, which was associated with epigenomic reprogramming of myeloid progenitor cells, although the enhanced innate immune responses declined significantly soon after the mice resumed a chow diet.^51^ Similarly, controlled caloric restriction promoted immuno‐metabolic reprogramming leading to protection against tuberculosis.^70^ In contrast, the urban lifestyles of city dwellers conferred a proinflammatory immune phenotype compared to rural dwellers; serum from urban dwellers induced reprogramming of innate immune cells to give higher TNF‐α production upon microbial restimulation in the in vitro model of trained immunity. This could be inhibited by the flavonoid apigenin, consistent with the importance of traditional, plant‐based diets in modulating immunological resilience.^71^ These researches together suggest that disease epidemiology can be changed by trained immunity.

Several studies suggested that there might be other signals that contribute to the development of trained immunity besides immunometabolic and epigenetic reprogramming of HSPCs and mature myeloid cells. These have provided further clues to the underlying mechanisms. For example, β‐glucan‐mediated heterologous protection was absent in IL‐1R^–/–^ mice, suggesting that IL‐1 signaling is crucial in β‐glucan‐induced trained immunity.^72^ Priming with hematopoietic cytokines GM‐CSF and IL‐3 followed by 6 days of resting resulted in increased TNF‐α production upon LPS restimulation in human monocytes.^73^ Monocytes trained with β‐glucan depended on the increased expression of IL‐32 to confer enhanced protection against *Leishmania braziliensis* infection.^74^ Sterile tissue injury can train the innate immune system, for example, in providing protection against subsequent infection in adult drosophila.^75^ The induction of trained immunity might also be modulated by circadian rhythm^76^ and in a sex‐dependent manner.^77^ Recently, it was reported that microRNA also participated in the regulation of trained immunity.^78^

The balanced fine‐tuning of innate immune responses is a regular job of immune systems, but most research has focused on the induction, rather than the regulatory down‐modulation, of trained immunity. Examples of antagonists acting against trained immunity are: IL‐37 reversed the immuno‐metabolic and histone posttranslational modifications of trained immunity in monocytes; this abrogated the host defense against infection by suppressing the secretion of inflammatory cytokines.^79^ IL‐38 impaired β‐glucan‐induced trained immunity by blocking the target for rapamycin (mTOR) signaling and preventing epigenetic and metabolic changes.^80^ Antagonists other than cytokines have been described. Hydroxychloroquine prevented the induction of epigenetic modifications needed for trained immunity.^81^ Preoperative exercise therapy triggered an anti‐inflammatory trained immunity phenotype via metabolic reprogramming and led to attenuation of liver injury and inflammation from ischemia in mouse models.^82^ Sterile tissue injury trained the innate immune system, providing protection against subsequent infection in adult drosophila.^75^ The excretory‐secretory products of *Fasciola hepatica* imprinted a long‐term anti‐inflammatory phenotype in myeloid cells.^83^ The induction of trained immunity might also be modulated by circadian rhythm,^76^ in a sex‐dependent manner,^77^ and by microRNA.^78^

Recently, it was shown that trained immunity could be inherited in a murine model. Male mice were infected with sublethal *C. albicans*, and the resistance and responsiveness against heterologous infection were observed in the offspring; transcriptional and epigenetic changes in bone marrow progenitors were found.^84^ These results provide evidence for the inheritance of trained immunity in mammals, consistent with the hypothesis that trained immunity is an evolutionarily conserved means of immune memory‐based enhanced preparedness for future challenges.

To sum up, the research suggests that different stimuli might activate different trained immunity programs, and our knowledge of the signaling pathways that are involved in trained immunity modulation is still increasing.

## 5 THE YIN‐YANG BALANCE OF TRAINED IMMUNITY

**FIGURE 4:**
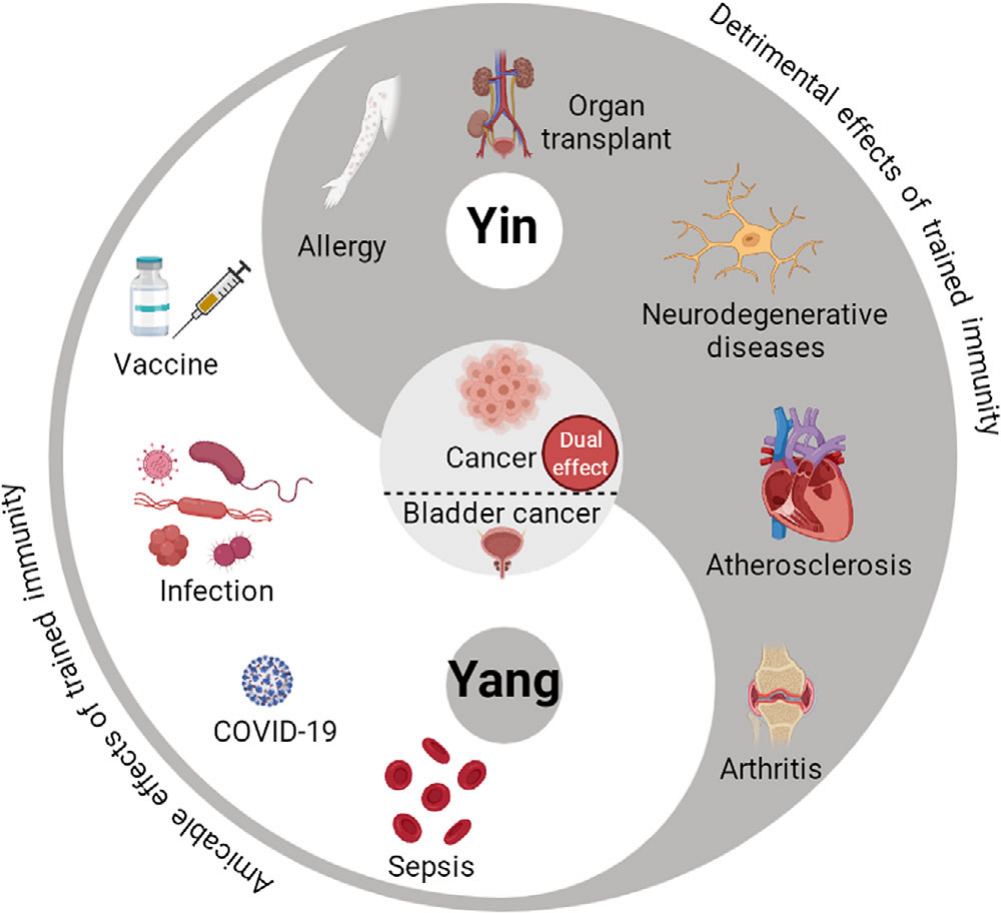
The Yin‐Yang balance of trained immunity. Trained immunity is a protective mechanism evolved to protect the host against infection and reinfection. However, a maladaptive effect of trained immunity can result in hyperinflammation, immune tolerance, or suppression that imbalance the immune systems. These compatible effects can be viewed as consistent with the Yin‐Yang theory of traditional Chinese philosophy, in which Yang represents the active, positive, and aggressive factors, whereas Yin represents the passive, negative, and inhibitory factors. The amicable (Yang) effects of trained immunity endow protection against infections such as COVID‐19, prevent sepsis, and can be utilized to optimize vaccination strategy. The detrimental (Yin) effects of trained immunity are associated with autoimmune and inflammatory disorders such as rheumatoid arthritis, atherosclerosis, neurodegenerative diseases and allergy, as well as contribution to allograft rejection in organ transplant surgery. The antitumor innate immune memory can hold both the Yang and Yin aspects: although BCG instillation induced trained immunity might be effective in treating bladder muscle cancer, the hyperactivated and chronic inflammation induced by trained immunity might also facilitate tumor growth. This figure was created with BioRender.com

Trained immunity can be regarded as a basic protective mechanism that has evolved to protect against infection and reinfection. However, a maladaptive induction of trained immunity might result in hyperinflammation, immune tolerance, or immune‐suppressive responses that reflect an imbalance in the immune systems. The contrary effects could be considered in the context of the Yin‐Yang theory of traditional Chinese philosophy, in which Yang represents active, positive, and aggressive factors, whereas Yin represents passive, negative, and inhibitory factors (Figure 4). The essence of Yin‐Yang theory is that everything has Yin and Yang aspects and their balance makes the world.

### 5.1 The amicable (Yang) effects of trained immunity

Neonates rely on the innate immune systems, supplemented with antibodies from breast milk, to combat infection. Subsequently, vaccines such as BCG, and encounters with nonpathogenic bacteria/viruses train the innate immune systems and enhance protection against both related and unrelated secondary infections. As a typical trained immunity‐inducing agonist, β‐glucan affords protection against heterologous pathogenic infections including *Staphylococcus aureus*,^50^ *Leishmania braziliensis*,^74^ *Pseudomonas aeruginosa*,^85^ and *M. tuberculosis*.^72^ Moreover, respiratory BCG vaccination protected mice against a lethal *S. Pneumoniae* infection, which was associated with preactivated alveolar macrophages.^86^ The accelerated clearance of invading pathogenic microorganisms by trained innate immunity is important. The earlier that the infection is eliminated or brought under control then the more the destructive effects of not only the microorganism but also of the immune responses are controlled. Persistent infection can lead to sepsis, which results in exaggerated systemic immune responses during which secondary organ damage is often fatal. Thus, optimal induction of trained immunity contributes to enhanced protection against both infection and immune damage.

In the early phase of the current coronavirus disease 2019 (COVID‐19) pandemic outbreak, the lack of a specific vaccine against the causative agent, severe acute respiratory syndrome coronavirus‐2 (SARS‐CoV‐2), led to renewed interest in trained immunity. Several epidemiological studies showed a possible association of BCG vaccination history with decreased COVID‐19‐related risks, suggesting BCG had provided some protection against COVID‐19.^87^, ^88^, ^89^ In addition, retrospective observational studies showed that a history of BCG vaccination was associated with decreased seroprevalence of anti‐SARS‐CoV‐2 IgG, reduced clinical symptoms, and decreased severity of disease in a cohort of health care workers.^90^ Similarly, previously influenza vaccination was associated with SARS‐CoV‐2 infection in a Dutch hospital, which was associated with a trained immunity program that boosted innate immunity against various viral stimuli and fine‐tuned the anti‐SARS‐CoV‐2 immune response.^91^ The BCG‐induced nonspecific effects against SARS‐CoV‐2 were suggested to be partially due to BCG‐trained immunity.^92^, ^93^, ^94^, ^95^, ^96^ More recently, single‐cell chromatin accessibility analyses of peripheral blood mononuclear cells showed that, compared to healthy volunteers, individuals who had recovered contained abundant TBET‐enriched CD16^+^ and IRF1‐enriched CD14^+^ monocytes consistent with trained and activated epigenomic states.^97^ In contrast to healthy individuals, recovered COVID‐19 patients had macrophages that showed profound and long‐lived reprogramming. The resulting robust S‐protein‐driven inflammasome activation and distinct epigenetic and gene expression signatures suggested that infection with COVID‐19 itself resulted in trained immunity.^98^ Several clinical trials are currently investigating the effectiveness of the BCG against COVID‐19 in multiple countries; these results should produce more definitive answers on the role of BCG against COVID‐19.^99^

In addition to nonspecific protective effects against infection, the BCG vaccine has notable antitumor activity. In 1976 it was reported to elicit a potent intratumoral cellular inflammatory response that somehow could induce tumor shrinkage after intravesical instillation as an adjunctive treatment for noninvasive cancer of bladder muscle.^100^ This immunotherapy was impressive, resulting in a decrease in the recurrence rates, and was the first successful treatment of this nature against a solid human tumor. Currently, BCG instillation is the gold standard therapy for noninvasive high‐ and medium‐risk bladder muscle cancer.^101^, ^102^ Although the precise mechanism is still unclear, trained immunity was shown to at least partly account for the heterologous beneficial effects. The mRNA levels of trained immunity markers IL‐1β and TNF‐α were increased in peripheral blood following the first instillation of BCG compared with the baseline levels, and these cytokines were significantly increased after the sixth instillation.^103^ Compared to healthy controls and untreated patients, monocytes isolated from patients after BCG instillation showed heightened TNF‐α responses upon LPS reexposure.^104^ In BCG instillation therapy, the activity of autophagy gene *ATG2B* was found to be correlated with the progression and recurrence of bladder cancer after BCG intravesical instillation therapy and regulated trained immunity via epigenetic reprogramming of monocytes.^105^ The epigenetic modifications and metabolic changes in innate immune cells during BCG instillations against bladder cancer warrant further investigation to generate a blueprint for superior ways of inducing trained immunity in a personalized approach to therapy in bladder cancer. The research may have broader implications for cancer therapy since a 60‐year follow‐up retrospective clinical trial showed that childhood BCG vaccination was associated with a lower risk of lung cancer development in the United States.^106^ In addition, β‐glucan treatment induced a CCR2‐dependent influx of monocytes to the pancreas that display trained immunity profile, and β‐glucan‐treated mice showed reduced tumor burden and prolonged survival in orthotopic models of pancreatic cancer.^107^

To sum up, optimal induction of trained immunity can lead to enhanced protection against infections and cancer. Through further research, novel therapeutic targets may become available to harness trained immunity to improve immune responses to vaccines and resistance to infections.

### 5.2 The detrimental (Yin) effects of trained immunity

Although the enhanced inflammatory responses induced by trained immune cells can impart enhanced protection against infections, trained immunity might also be detrimental. In principle, the excessive or long‐term activation of innate responses induced by trained immunity might lead to hyperinflammation or immunosuppression during chronic diseases. An increasing number of studies provide evidence that reprogramming in trained immunity may play a part in maintaining diverse disorders. Herein, we summarize the diseases linked to abnormal changes in innate memory programs.

Rheumatoid arthritis is a chronic inflammatory disease leading to joint destruction. It was found that synovial fibroblasts from patients with rheumatoid arthritis were activated and produced high levels of disease‐promoting molecules, which autonomously drove joint inflammation and destruction. Epigenetic modulation was found to be responsible for the changes in gene expression and to shape the pathogenic phenotype. Importantly, after lengthy cellular exposure to inflammatory stimulation, the cells were imprinted with activated states that became independent of the activating stimulus and persisted even in the absence of the stimulus.^108^ Consistent with this phenomenon, inhibition of glycolysis by 2‐deoxyglucose^109^ and the inhibition of trained immunity by the inhibitor I‐BET151 (which will be discussed in the next section)^110^ have the capacity to reduce chronic inflammation and disease severity in rheumatoid arthritis.

Atherosclerosis is a chronic inflammatory disease of the arteries and is the underlying cause of about 50% of all deaths in westernized society. As mentioned above, oxLDL, an endogenous atherogenic stimulus, is one of the inducers of trained immunity.^45^, ^46^ Chronic stimulation by oxLDL could be a contributory factor to atherosclerosis. In observational studies, monocytes from atherosclerosis patients exhibited a trained immunity phenotype with enhanced inflammatory cytokine secretion and increased glycolytic activity, compared with healthy volunteers.^61^, ^111^ In addition, in a murine model, it was found that subclinical endotoxemia, which was often seen in humans with chronic inflammation, aggravated atherosclerosis through programming monocytes into a nonresolving inflammatory state.^112^ Consistent with this phenomenon, partial inhibition of glycolysis by 3PO reduced atherosclerosis development in a murine model.^113^ Moreover, progesterone suppressed oxLDL‐induced trained immunity in macrophages, showing the potential in treating atherosclerotic cardiovascular diseases.^114^

The exposure of microglia, the innate immune cells of the brain, to inflammatory stimuli can induce a long‐lasting effect of immunity. This was demonstrated by showing that when the microglia encountered a subsequent stimulus, they produced an increased inflammatory response. This phenomenon, which was called “microglial priming,” is consistent with the concept of trained immunity.^115^ Similar to experimental models of trained immunity, 4 weeks after infection of mice with *Salmonella typhimurium*, microglia had greater immune reactivity and exaggerated local inflammatory responses to subsequent stimulation with LPS.^116^ Recently, rapamycin was shown to suppress the trained immunity‐like responses of microglia through targeting the mTOR/HIF‐1α signaling pathway,^117^ providing novel insights into current therapeutic strategies in neurodegenerative diseases.

Allergy arises from hypersensitivity of the immune system to typically harmless substances in the environment. Compared with nonallergic children, allergic children showed exaggerated innate responses to TLR ligands at birth,^118^ and cord blood monocytes of infants who subsequently developed food allergies produced higher amounts of inflammatory cytokines IL‐1β, IL‐6, and TNF‐α in response to LPS.^119^ These studies suggest that unbalanced trained immunity is associated with allergy and the induction of an optimized state of trained immunity might be beneficial. Prior BCG vaccination has been found to hinder allergic sensitization and the development of increased airway reactivity in an animal model of allergic airway disease,^120^ and a large randomized clinical trial is being conducted to study whether BCG vaccination in healthy neonates protects against allergic diseases.^121^

Organ transplantation rejection occurs when transplanted tissue is rejected by the recipient's immune system and destroyed. Although it has long been thought that only adaptive immunity mediated this process, the roles of innate immune cells and trained immunity are beginning to be uncovered. Trained macrophages have been proposed to upregulate costimulatory molecules and proinflammatory cytokines to enhance the potency of graft reactive immune responses and organ transplant rejection.^122^ In 2018, a novel nanomedicine targeting trained immunity (which will be discussed in the next section) showed potential application for transplantation therapeutics.^123^

Although the trained immunity induced by BCG instillation might be effective in treating bladder muscle cancer, the induction of trained immunity is a double‐edged sword in cancer. Hyperactivated and chronic inflammation might also facilitate tumor growth. Recently, it was found that an oncogenic mutation in monocyte/macrophage cells induced maladaptive activation of trained immunity leading to hyperinflammation and pathological tissue damage in an inflammatory myeloid neoplasm disease.^124^ Thus, further research is warranted to better understand the role of trained immunity in cancer, to harness its therapeutic potential while suppressing its tumor‐promoting effect.

In summary, an uncontrolled hyperactivated immune response resulting from trained immunity could be implicated in a variety of diseases with an autoimmune component. Thus, modulating trained immunity to achieve long‐term therapeutic benefits may prove to be a valuable strategy for attenuating anomalous reactions in chronic inflammatory diseases, autoimmunity, and a range of other trained immunity‐related pathologies.

### 5.3 The compatibility of effects of trained immunity with the Yin‐Yang concept

Actually, in traditional Chinese philosophy, Yin and Yang are relative properties, not invariable, and might be transformed into each other under certain conditions. From an immunologist's point of view, an individual's immune system encounters numerous infectious pathogens throughout the lifespan. These interactions can have a long‐lasting impact on the immune system's functionality. At an individual level, the enhanced trained immunity might contribute to more efficient clearance of infection but also might induce autoimmune diseases; similarly, the inhibition of trained immunity might ease local inflammation but also impair the innate immune defense against infection. These contrasting effects make trained immunity an attractive but challenging therapeutic target.

Although trained immunity is a durable process, the immune memory is not a permanent one and presents opportunities for therapeutic manipulation. However, much effort is still required to decipher how trained immunity influences the progression of individual diseases in order to develop effective but safe therapies.

## 6 POTENTIAL CLINICAL IMPLICATIONS OF MODULATING TRAINED IMMUNITY

In a murine model of systemic sclerosis, training immunity with LPS alleviated inflammation and fibrosis, whereas BCG training exacerbated disease.^125^ Thus, optimization of the Yin and Yang balance of trained immunity can improve resistance to diseases but in contrast, an unbalancing of Yin and Yang effects might be deleterious. Accordingly, any clinical reprogramming of trained immunity, whether with established or with novel agents targeting the pathways, must be approached with caution.^126^ Clinical and preclinical studies in trained immunity‐targeting therapies/vaccines are summarized in Table 1.

**TABLE 1:** Clinical and preclinical studies in trained immunity‐targeting therapies/vaccines

Agents	Diseases	Mechanisms	References
BCG	COVID‐19/infectious diseases	Accelerated clearance of invading pathogenic microorganisms by heterologous trained innate memory	^28^, ^29^, ^90^
BCG	Bladder cancer	The inflammatory milieu induced by BCG instillation, and trained immunity‐regulated adaptive immunity beneficially in the antitumor responses	^100^, ^106^
BCG	Allergy	Skew away from Th2 responses and optimization of inflammatory cytokines	^120^, ^121^
MTP_10_‐HDL	Melanoma	Durable anticancer innate immune response by stimulating the production of trained myeloid cells and their resulting influx into the tumor microenvironment	^130^
2‐Deoxyglucose/I‐BET151	Rheumatoid arthritis	Inhibition of glycolysis/suppression of inflammatory cytokines	^109^, ^110^
3PO/progesterone	Atherosclerosis	Inhibition of glycolysis/suppression oxLDL‐induced trained immunity	^113^, ^114^
Rapamycin	Neurodegenerative diseases	Suppression of mTOR/HIF‐1α signaling mediated trained immunity	^117^
mTORi‐HDL	Organ transplantation rejection	Inhibition of macrophage aerobic glycolysis and epigenetic modifications underlying trained immunity, and promotion tolerogenic expansion of regulatory T cells	^123^
Antibodies targeting IL‐1β and GM‐CSF	Rheumatoid arthritis, type 2 diabetes, asthma, etc.	Inhibition of inflammation, the contributions of trained immunity remain to be explored	^131^, ^132^, ^133^
AS03‐adjuvanted H5N1 influenza vaccine	Heterologous viral infection	Diminished H3K27ac in monocytes and impaired cytokine responses to Toll‐like receptor stimulation	^137^

### 6.1 Nanoimmunotherapy

Nanotechnologies are undergoing intensive development for potential application in many areas including nanoparticle‐based medicines.^127^ Although nanomedicines targeting myeloid cells have shown promise in several areas,^128^, ^129^ the mechanisms of action are not fully understood, which hinders further application. Nevertheless, potential applications continue to be explored in murine models. In an experimental heart transplantation mouse model, short‐term treatment with nanobiologics targeting mTOR inhibitor as immunotherapeutic agents had a favorable outcome. The treatment averted the development of the macrophage aerobic glycolysis and epigenetic modifications underlying trained immunity. The resultant tolerogenic regulatory macrophages promoted tolerogenic expansion of regulatory CD4 T cells, prevented alloreactive CD8 T cell immunity, and enhanced allograft survival.^123^ Importantly, this therapeutic approach modified not only target graft‐infiltrating myeloid cells but also their progenitors in the bone marrow. Thus, nanomedicine might be used to inhibit trained immunity induction and promote immunological tolerance in clinical applications. Building on the evidence that the anticancer effects of BCG may be, at least partly, accounted for by induction of trained immunity by MDP,^30^, ^39^ the potential of nanoparticles is being evaluated.^130^ Treatment of mice with a leading nanobiologic candidate, MTP_10_‐HDL, inhibited tumor growth and activated HPSCs. The effects were mediated by the trained immunity‐induced myelopoiesis caused by epigenetic reprogramming of multipotent progenitors in the bone marrow. In nonhuman primates, this nanobiologic accumulated in hematopoietic organs and displayed a favorable safety profile.^130^ This study may pave the way for exploiting trained immunity induction in an in vivo immunotherapy approach.

### 6.2 Antibody immunotherapy

Because cytokines mediate the development and expression of trained immunity, antibodies against them and their pathway elements should be effective in inhibiting or enhancing trained immunity. In fact, before the concept of trained immunity had emerged, a neutralizing monoclonal anti‐IL‐1β antibody had already shown efficacy in clinical trials against several inflammatory diseases including rheumatoid arthritis, type 2 diabetes, and acute stroke.^131^ More recently, a randomized, double‐blind Phase III trial in atherosclerosis showed that canakinumab, a therapeutic monoclonal antibody targeting IL‐1β led to a significantly lower rate of recurrent cardiovascular events than a placebo.^132^ Similarly, antibody‐based therapies targeting GM‐CSF or its receptors have been tested in clinical and preclinical studies with promising results, including in rheumatoid arthritis, plaque psoriasis, and asthma.^133^ However, the contributions of trained immunity in these studies remain to be explored.

### 6.3 Vaccines targeting trained immunity

Conventional vaccines are primarily intended to target specific pathogens by inducing long‐term antigen‐specific adaptive immune memory. Innate immune systems participate in the establishment of adaptive immunity by expressing pattern recognition receptors that bind to components of pathogens, trigger expression of costimulatory molecules and inflammatory cytokines, and tune the antigen presentation process. We now know that the innate immune system can also be trained to provide long‐lasting innate memory responses to homologous and heterologous secondary insults. In consequence, trained immunity‐based vaccines might be either designed to modify trained immunity per se or designed for integral codelivery to optimize the supportive interactions with antigen‐specific acquired immunity—an adjuvant effect. An example is available in a mouse model of bladder cancer therapy. Compared with wild‐type BCG, a recombinant BCG expressing pertussis toxin could induce higher levels of trained immunity marker TNF‐α in bladders and conferred longer survival times,^134^ and more IL‐6 was produced in an in vitro assay of stimulated human blood leukocytes.^135^ Recently, a recombinant BCG expressing a STING agonist showed enhanced antitumor efficacy by triggering trained immunity remodeling.^136^ Another example is a squalene‐based adjuvant that contains α‐tocopherol, AS03, which facilitates robust innate immune responses. As an adjuvant to an influenza vaccine, it enhanced both chromatin accessibility at antiviral response loci and resistance to infection with heterologous viruses.^137^ Additionally, the nano‐based immune‐therapeutics targeting the metabolic and epigenetic responses that were discussed in the previous sections, have the potential to fine‐tune specific and nonspecific effects of vaccines. Such approaches might be used to improve the efficacy of current vaccines by adjuvating or might serve to directly enhance long‐term nonspecific effector responses of innate immune cells against heterologous pathogenic microorganisms.

## 7 SUMMARY AND OUTLOOK

Trained immunity is a recently described property of innate immune cells that undergo long‐term functional reprogramming in response to endogenous and exogenous insults. The cells acquire long‐lasting alterations to their capacity to mount inflammatory responses against a second stimulation. This novel concept of memory that is resident in the innate immune system cells and the Yin‐Yang duality of the effects have opened new perspectives on the control of infection and inflammation. Accordingly, opportunities arise to devise novel approaches for vaccine development, and precision medicine with tailored individual immunomodulators for either enhancement/Yang or suppression/Yin of selected responses.

Metabolic and epigenetic reprogramming are the central mechanisms underlying trained immunity. The pathways and mechanisms involved are beginning to be clarified, and attaining a thorough understanding of the molecular mechanisms that modulate the interplay between innate and adaptive immunity remains a priority. Precise mechanistic descriptions will provide the developmental groundwork for specific therapies targeting key cell populations and their progenitors. The high specificity inherent in nanomedicines is already finding potential applications in trained immunity modulation and further research is likely to dramatically expand the number of clinical studies.

In particular, we may expect the future research directions for trained immunity to be focused on the following aspects. First, the knowledge gaps still exist in our understanding of mechanisms behind the epigenetic and metabolic branches. Unraveling the elaborate cellular and molecular mechanisms of trained immunity will be critical for devising novel approaches to the development of trained immunity‐based immunotherapies. Recently, the comprehensive view of cellular transcriptional signatures of trained immunity induced by different stimuli was identified at single‐cell resolution,^138^ and an integrative genomics approach identified several genetic loci^139^, ^140^; future studies are warranted to further investigating their functionality. Second, the available range of epigenetic modifiers is limited. More candidates must be investigated for incorporation into the trained immunity‐targeting nanomedicine therapies. Notably, the bromodomain and extraterminal (BET) domain family of proteins is increasingly recognized as able to target inflammatory gene expression by interfering with acetylated histones recognition.^141^, ^142^ A small molecular histone mimic BET inhibitor I‐BET151 suppressed fungal pathogens *C. albicans*‐induced trained immunity and reduced chronic inflammation in arthritis.^110^, ^143^ Further research of other BET inhibitors targeting trained immunity is warranted. Third, the question remains to be answered whether short‐term induction/inhibition of molecules that mediate trained immunity is sufficient to write or remove the associated epigenetic markers and programming of trained immunity, or only provides dynamic interference? Fourth, Dr. Netea proposed a strategy to design next‐generation vaccines termed “amplified” vaccines, which would contain classical vaccine components and metabolic/epigenetic modulators with the capacity to amplify the function of immunity.^144^ Such approaches might not only improve the nonspecific trained immunity against different types of pathogens but also potentially enhance the antigen‐specific adaptive immune responses, as we indicated in previous sections. The potential of this novel vaccine strategy is waiting to be tested in the coming years. Fifth, the recent finding of the intergenerational inheritance property of trained immunity suggests a novel approach to immunotherapy against hereditary diseases^84^; more studies are needed to investigate the mechanisms mediating this phenomenon at the immunological and molecular levels.

In conclusion, as a recently described property of innate immune cells, trained immunity added a layer of complexity to the previous understanding of immune systems. The Yin and Yang aspects of trained immunity provide us with an opportunity to treat inflammatory, infectious, and autoimmune diseases by optimizing innate immune memory and improving the efficacy of vaccination. We envision that more efforts will be focused on developing novel modulators/vaccines targeting trained immunity in the future.

## CONFLICT OF INTEREST

The authors confirm that there are no conflicts of interest.

## ETHICS APPROVAL

No ethical approval was required for this study.

## AUTHORS' CONTRIBUTIONS

ZDH and XYF conceived, designed and wrote the manuscript, XYF, DBL and SHL edited the manuscript with conceptual advice.

## Data Availability

Not applicable.
